# Surgical treatment outcomes of the Ilizarov and internal osteosynthesis methods in posttraumatic pseudarthrosis of the tibia—a retrospective comparative analysis

**DOI:** 10.1186/s13018-020-01697-4

**Published:** 2020-05-19

**Authors:** Łukasz Szelerski, Sławomir Żarek, Radosław Górski, Karol Mochocki, Ryszard Górski, Piotr Morasiewicz, Paweł Małdyk

**Affiliations:** 1 Department of Orthopedics and Musculoskeletal Traumatology, Medical University of Warsaw, Lindleya 4, 02-005 Warsaw, Poland; 2 Department and Clinic of Orthopaedic and Traumatologic Surgery, Wroclaw Medical University, Borowska 213, 50-556 Wroclaw, Poland

**Keywords:** Ilizarov method, Aseptic pseudarthrosis, Tibial nonunion, External fixator

## Abstract

**Introduction:**

This study compared surgical treatment outcomes of the Ilizarov and internal osteosynthesis methods in posttraumatic pseudarthrosis of the tibia.

**Material and methods:**

In a retrospective comparative study, 75 patients were treated with the Ilizarov technique for aseptic posttraumatic pseudarthrosis of the tibia in the period 2000–2016. We compared them with the 51 patients from the control group, treated for tibial bone union disturbances using internal osteosynthesis methods, i.e., internal-fixation plates and intramedullary nails. The study groups were compared in terms of the rates of union, time to union, and the baseline-to-postoperative difference in lower leg deformity.

**Results:**

Union rate in the Ilizarov group was 100% and the control group was 51.92% (*p* < 0.001). The median time to union suggests that patients from the Ilizarov group needed a shorter time to achieve bone union (203.00 days vs. 271.00 days) (*p* = 0.091). The effect size in the Ilizarov group was larger both in terms of reducing both limb deformity and shortening (it is worth noting, however, that the Ilizarov treatment was used in patients with higher baseline values of both these parameters). We observed no significant difference in terms of time to union between the group of patients with at least one risk factor for disturbance in fracture healing and the group with no risk factors. The following risk factors were considered: diabetes mellitus, corticosteroid therapy, smoking, alcohol dependence, and advanced lower-extremity vascular disease (*p* = 0.827).

**Discussion:**

Our study demonstrated a high effectiveness of the Ilizarov method in the treatment of aseptic posttraumatic pseudarthroses of the tibia. The Ilizarov method seems to be worth considering in all cases where either the patient or the nature of injury is associated with additional risk factors and whenever there is a need for leg deformity correction or leg elongation.

## Introduction

Posttraumatic pseudarthrosis of the tibia is a persisting nonunion observed 6–8 months following the tibial fracture [1]. A lack of callus formation between bone fragments results in pain, deformity, and pathological mobility in the limb. The estimated proportion of disturbed tibial union is 2.5–11% of all tibia fractures, which is the highest percentage in all long bones [1–4].

There have been a number of reported factors that predispose to bone healing disturbances. These factors include inadequate bone-fragment stabilization, impaired bone perfusion due to periosteal damage, associated soft tissue loss, and wound contamination (resulting in injury site infection). Moreover, the groups of patients at a higher risk of disturbed bone healing also include diabetics, patients undergoing corticosteroid treatment, smokers, and patients with alcohol dependence [5, 6].

Tibia pseudarthroses represent a wide spectrum of diagnoses that can be divided into several subtypes. Based on the extent of pathological mobility, pseudarthroses can be classified as stiff or mobile. The amount of bone fragment perfusion leads to hypertrophic (well-perfused) or atrophic (inadequately perfused) pseudarthroses. Another subgroup constitutes infected pseudarthroses, where a superimposed infection is an additional factor that worsens the prognosis. Due to the variety of pseudarthrosis types (presented above), there is no single universal surgical technique, and each case must be considered individually [7]. Despite the progress in surgical methods and the development of novel implants, treatment of bone nonunion remains a challenge for orthopedic surgeons. Extended therapy, which can last months or even years, is expensive and adversely affects the patients’ professional and personal life [8, 9].

The available relevant literature contains predominantly articles on the treatment of infected pseudarthroses of the tibia, with much fewer accounts focusing on the treatment of aseptic pseudarthroses of the tibia [3, 4, 10–13]. There are no comprehensive reviews assessing the treatment of aseptic pseudarthroses of the tibia in which the technique of Ilizarov osteosynthesis would be compared with that of internal osteosynthesis.

The purpose of our study was to assess the treatment outcomes in patients with aseptic posttraumatic pseudarthrosis of the tibia treated with the Ilizarov method and to compare them with the outcomes of treatment via internal osteosynthesis. The Ilizarov method has been used at our center for over 30 years. Over this period, the technique has been employed in the treatment of extensive fractures with soft tissue loss, bone nonunion, and in other indications.

## Material and methods

**Fig. 1:**
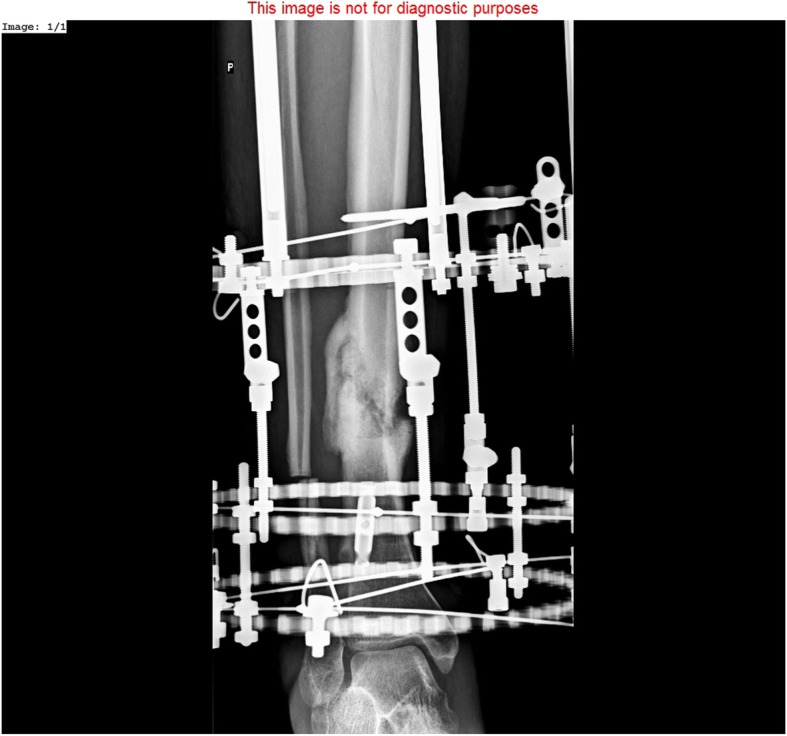
Hypertrophic pseudarthrosis of distal tibia stabilized by Ilizarov external fixator

Our analysis included 75 patients, who had been treated with the Ilizarov technique for aseptic posttraumatic pseudarthrosis of the tibia in the period 2000–2016 (Fig. 1). The inclusion criteria were aseptic pseudarthrosis of the tibia; fully accessible, complete patient’s medical and radiographic records; and follow-up at least 3 years or more after treatment. The exclusion criteria included infection at the site of bone injury and a bone tissue defect of > 1 cm. Moreover, patients with congenital crural pseudarthrosis, which constitutes a separate diagnosis, were also excluded from the analysis. The study was approved by the Institutional Local Review Board

Most of our patients suffered from closed tibial fractures occurred during car accident or falling down. Some of them had open tibial fracture with skin lesion < 1 cm (Gustilo-Anderson I). Patients with II and III grade (Gustilo-Anderson) open fracture usually are treated with Ilizarov external fixator primarily and so were excluded from the research. Unfortunately, in many cases, patients from other hospitals are sent without detailed information about their previous treatment. Mean age in the Ilizarov group was 45.1 years (range 10–84)

The pseudarthroses located at the proximal or middle thirds of the tibia were treated with an Ilizarov external fixator consisting of four rings fixed with Kirschner wires and Schanz screws. The pseudarthroses located at the distal third of the tibia were stabilized with a three-ring apparatus fixed with Kirschner wires and Schanz screws. Hypertrophic pseudarthroses were compressed or treated with neutral stabilization. In atrophic pseudarthroses, a small incision was made to decorticate (“scarify”) the surfaces of adjacent bone fragments, followed by stabilization of the pseudarthrosis with an Ilizarov fixator. All patients underwent resection of a 0.5–1-cm-long fibular segment.

Upright mobilization was introduced on postoperative day 1, and patients were taught to ambulate with the help of forearm crutches and encouraged to bear full weight on the operated limb. Follow-up assessments, including radiography, were initially conducted every 2 weeks and, subsequently, every 4 weeks. The fixator was removed following bone union (as evidenced by the presence of three out of four cortices and trabecular bridging). Following the removal of the fixator, some patients were fitted with a knee or crural brace, depending on the location of the pseudarthrosis. Four weeks later, full weight-bearing was allowed.

Our study evaluated the time required to achieve bone union and the extent of improvement in leg deformity and shortening compared to these parameters at baseline. The incidence and type of treatment-related complications was assessed, along with the effect of complications on treatment outcome. The findings were presented in the form of Association for the Study and Application of Methods of Ilizarov (ASAMI) bone scores and ASAMI functional scores [14, 15].

**Fig. 2:**
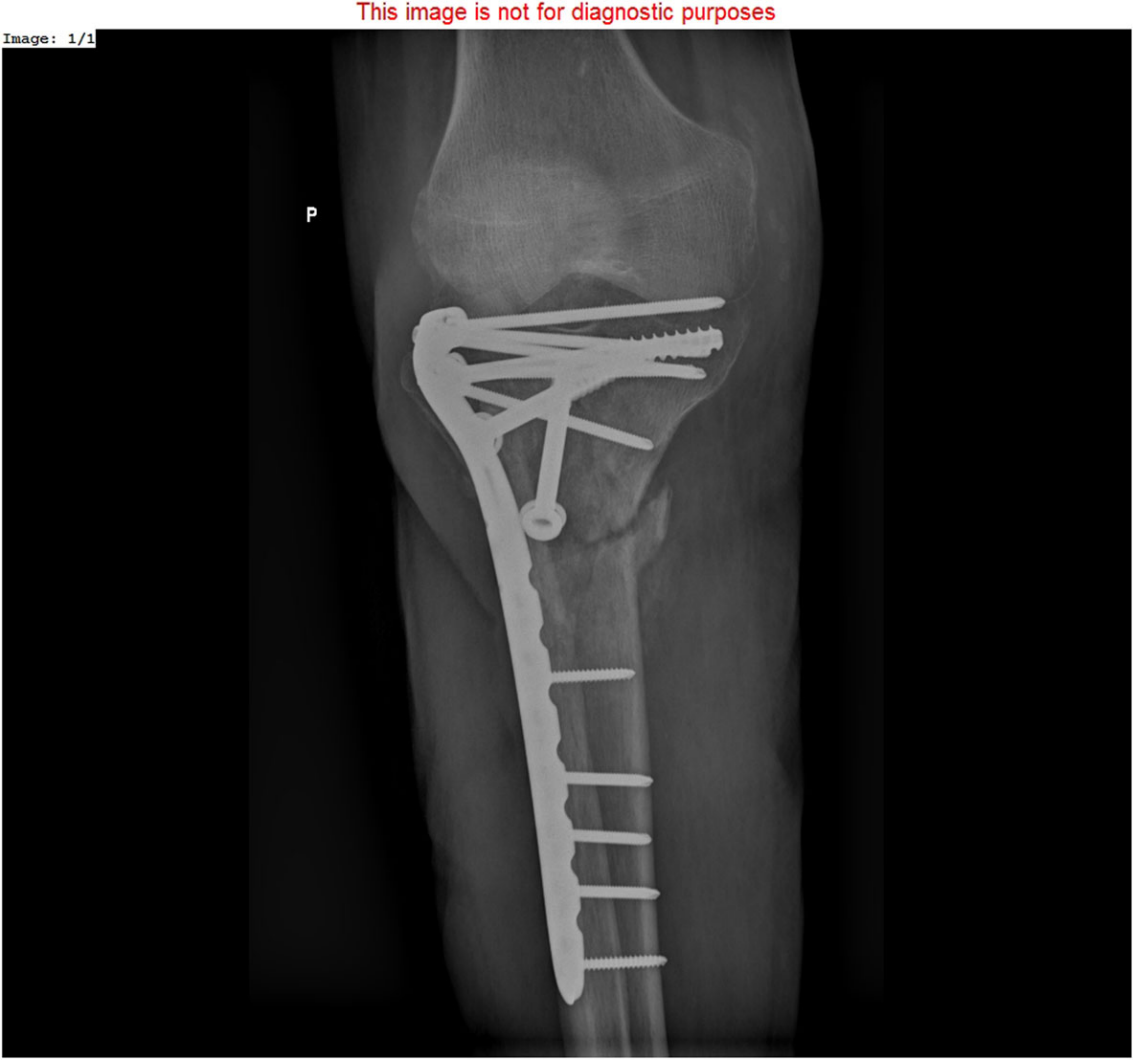
Nonunion of proximal tibia treated with locking compression plate and cancellous bone screws

**Fig. 3:**
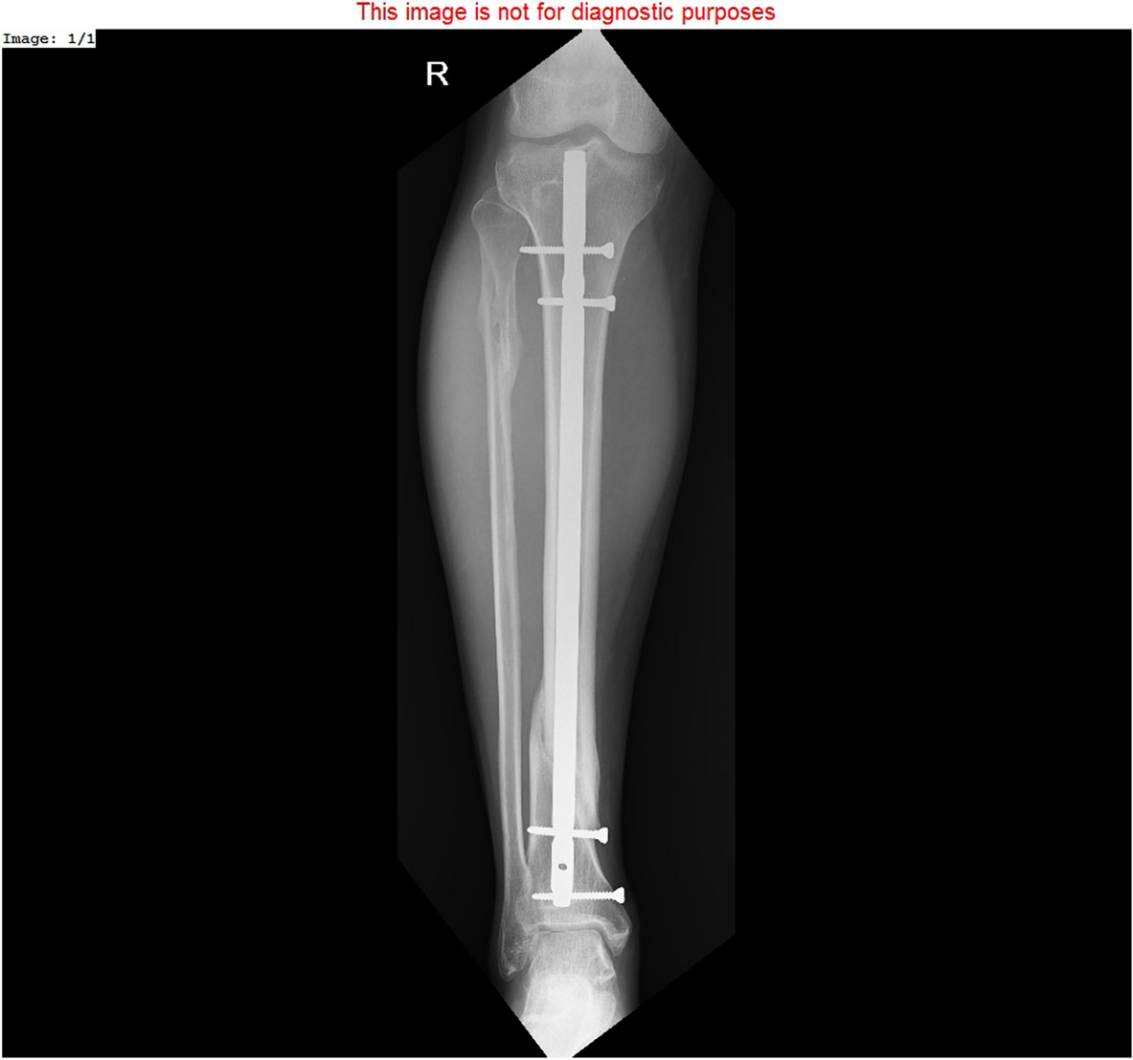
Bone union after treatment of hypertrophic tibia nonunion with reamed intramedullary nail

The 51 patients from the control group were treated for tibial bone union disturbances using internal osteosynthesis methods, i.e., internal-fixation plates and intramedullary nails (Figs. 2 and 3). These procedures were conducted by a different, experienced surgical team. Mean age in the control group was 40.4 years (range15–70).

The plate fixation technique involved trimming of the bone ends and the use of locking compression plates (LCPs). Bone defects were filled with autogenous iliac-wing bone grafts or allogenic bone grafts subjected to radiosterilization.

In patients originally treated with intramedullary nails, the initial implant was removed, the medullary canal was reamed, and a new intramedullary nail of a larger diameter was inserted. The operative technique depended on the location of the pseudarthrosis. Diaphyseal pseudarthroses were treated with intramedullary nails, whereas epiphyseal pseudarthroses were treated with plate fixation.

Postoperative mobilization was initiated on day 1 and involved active and passive exercises of the knee and ankle joints. Weight-bearing was initiated approximately 6 weeks after surgery in individuals who showed radiographic evidence of healing. Follow-up assessments, including radiography, were initially conducted every 2 weeks and, subsequently, every 4 weeks.

The study groups were compared in terms of the rates of union, time to union, and the baseline-to-postoperative difference in lower leg deformity.

The statistical analysis for testing the proposed hypotheses was conducted with STATISTICA 13.3 software. This software was used for descriptive statistics; the Shapiro-Wilk test was used to evaluate the normality of distribution of all quantitative parameters; frequency analysis was also conducted. Subsequently, the Mann-Whitney *U* test or Kruskal-Wallis test (ANOVA) was used to calculate differences between groups (due to a skewed distribution of data and disproportions in sample size between the individual subgroups). The Wilcoxon signed-rank test for paired samples was used for repeated measurements; potential correlation was assessed with Spearman’s rank correlation coefficient (*rho*). The chi-square test was used to compare the variables expressed as percentage values.

The level of statistical significance was adopted at *α* = 0.05; however, *p* values between 0.05 and 0.1 were interpreted as showing a statistical trend towards significance [16].

## Results

### Experimental group—patients treated with an Ilizarov external fixator

In order to assess any differences in time to union in the hypertrophic (*n* = 58) and atrophic (*n* = 17) pseudarthrosis subgroups treated with Ilizarov external fixation, the Mann-Whitney U test was used, yielding significant results (*Z* = − 2.31; *p* = 0.021; *η*^2^ = 0.07).

**Fig. 4:**
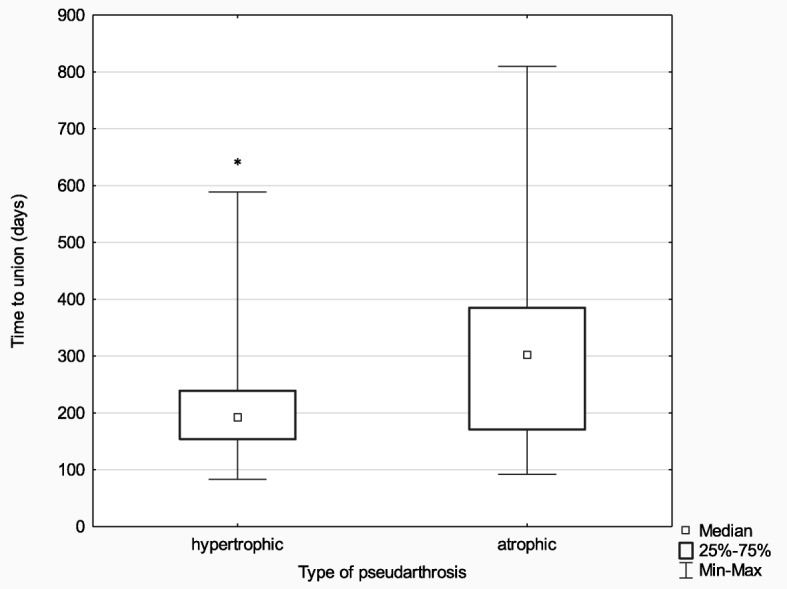
Median time to union in the hypertrophic and atrophic pseudarthrosis subgroups; *p ≤ 0.05

The median time to union was significantly shorter in the hypertrophic pseudarthrosis group (192.0 days) than in the atrophic pseudarthrosis group (301.0 days). More detailed data are presented in Fig. 4.

**Fig. 5:**
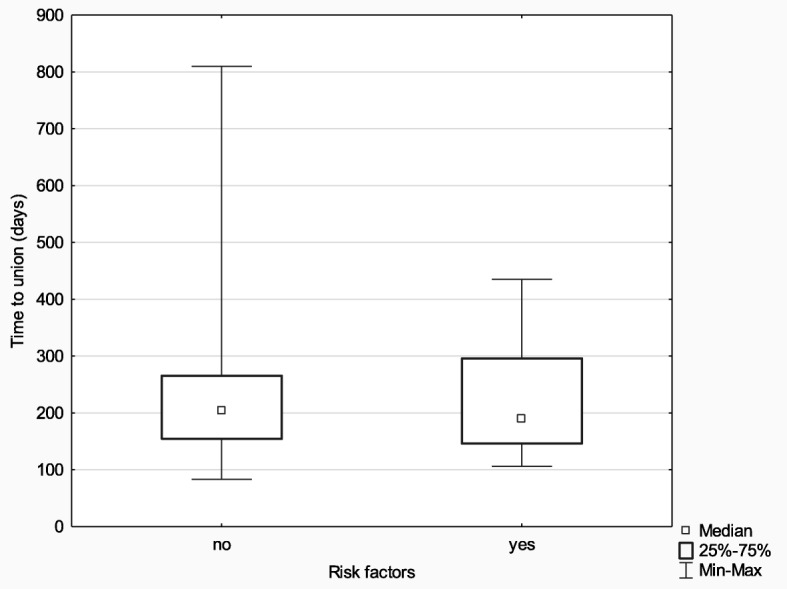
Median time to union in the subgroups with and without risk factors

We also analyzed the differences in time to union in the subgroup of patients (*n* = 15) with at least one risk factor for disturbances in fracture healing. The following risk factors were considered: diabetes mellitus, corticosteroid therapy, smoking, advanced lower extremity vascular disease, and alcohol dependence. This subgroup was compared with the subgroup with no additional risk factors (*n* = 60). Also, in this case, the results were analyzed with the use of the Mann-Whitney *U* test; though this time, analysis results were not statistically significant (*Z* = 0.22; *p* = 0.827; *η*^2^ < 0.01), suggesting a lack of relationship between the analyzed variables. More detailed data are presented in Fig. 5.

**Fig. 6:**
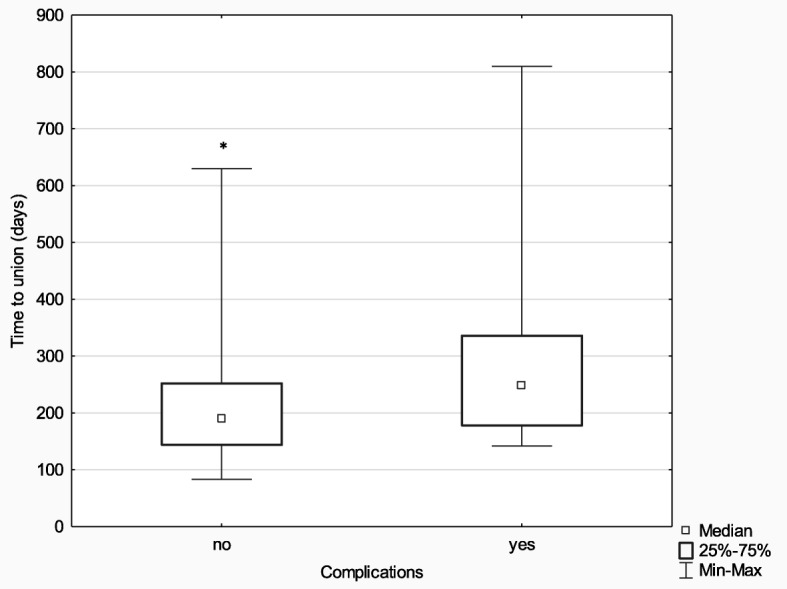
Median time to union in the subgroups with and without postoperative complications; *p ≤ 0.05

The differences in terms of time to union between the subgroup with treatment complications that required hospitalization (*n* = 22) and the subgroup with no complications (*n* = 53) were assessed with the use of the Mann-Whitney *U* test, which yielded statistically significant results (*Z* = − 2.15; *p* = 0.032; *η*^2^ = 0.06), suggesting a shorter median time to union in the no-complication subgroup (189.00 days vs. 248.50 days). More detailed data are presented in Fig. 6.

Before comparing the subgroups of Ilizarov patients treated with the closed and open Ilizarov method, we evaluated the two subgroups in terms of possible differences in baseline limb deformity and limb shortening. The results of both Mann-Whitney *U* tests showed no statistical significance (*Z* = 0.32; *p* = 0.747; *η*^2^ < 0.01 and *Z* = − 0.35; *p* = 0.729; *η*^2^ < 0.01, respectively), which demonstrated that the compared subgroups did not differ in terms of these parameters. Subsequently, to compare the effects of treatment via the closed (*n* = 47) and open (*n* = 28) Ilizarov method, the parameters of limb deformity and shortening before and after surgery were analyzed with the Wilcoxon signed-rank test for matched pairs. The postoperative measures of both limb deformity and limb shortening significantly improved in comparison with their baseline values in both analyzed subgroups (limb deformity: closed method *Z* = 5.38, *p* < 0.001, *r* = 0.55; open method *Z* = 4.46, *p* < 0.001, *r* = 0.60) (limb shortening: closed method *Z* = 5.19, *p* < 0.001, *r* = 0.54; open method *Z* = 3.82, *p* < 0.001, *r* = 0.51).

Effect sizes were comparable in both subgroups, with slightly larger effect sizes observed in terms of limb deformity reduction and following the open method; on the other hand, the closed method produced slightly better effects in terms of baseline limb shortening (these results are probably of low clinical value, as the observed differences were very small).

### A comparison between the experimental and control groups

**Fig. 7:**
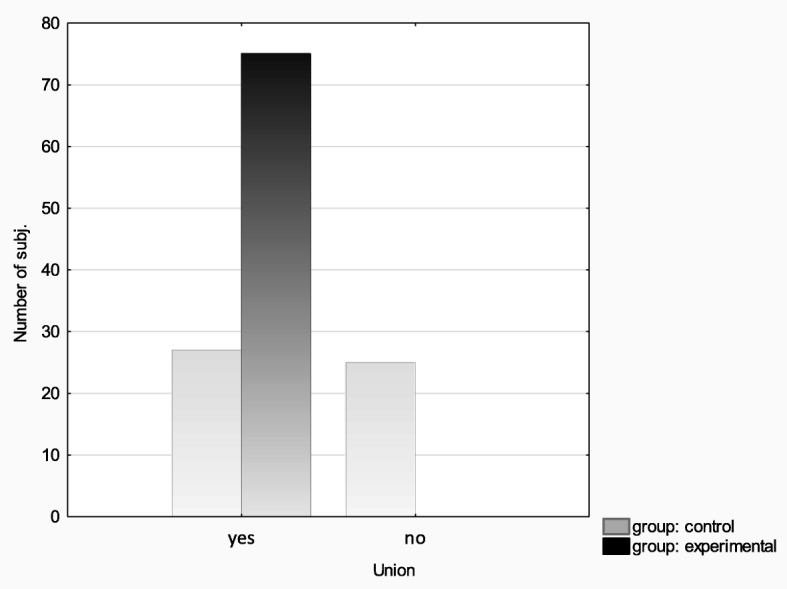
Union incidence in the experimental and control groups

The experimental and control groups were compared in terms of achieved bone union. The chi-square test was used in order to compare the rates of union in the experimental and control groups, yielding *χ*^2^(1) = 44.90, *p* < 0.001, which demonstrates a significantly higher proportion of patients with achieved bone union in the Ilizarov group (100% vs. 51.92%). See the bar graph (Fig. 7) below.

**Fig. 8:**
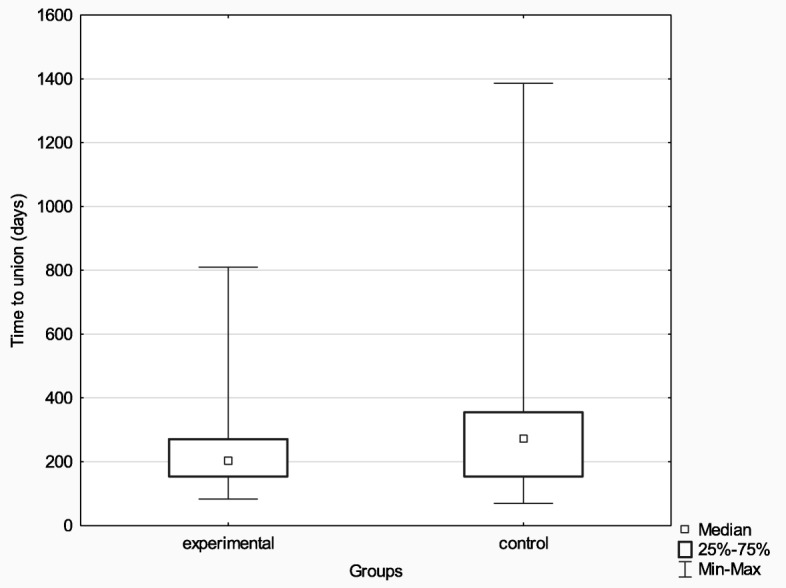
Median time to union in the experimental and control groups

The experimental (*n* = 75) and control (*n* = 27) groups were compared in terms of the median time to union with the use of the Mann-Whitney *U* test. The results were borderline significant (*Z* = − 1.69, *p* = 0.091, *η*^2^ = 0.03), which suggests that patients from the Ilizarov group needed a shorter time to achieve bone union (203.00 days vs. 271.00 days). Nonetheless, this conclusion should be considered circumspectly and verified in a study with a larger population. More detailed data are presented in Fig. 8.

### The achieved correction stratified by treatment method

The subgroups undergoing each of the two evaluated treatment methods were initially compared in terms of the baseline measures of limb deformity and limb shortening. In both respects, the Mann-Whitney *U* test yielded significant results (*Z* = 3.13, *p* = 0.002, *η*^2^ = 0.08 for limb deformity; *Z* = 3.21, *p* = 0.001, *η*^2^ = 0.08 for limb shortening), which indicates that the baseline values of both of these parameters were significantly greater in the Ilizarov group.

Subsequently, in order to analyze the effects of treatment depending on the method (Ilizarov external fixator (*n* = 75) and internal osteosynthesis (classic method) (*n* = 52)), the above parameters (limb deformity and shortening before and after treatment) were analyzed with the Wilcoxon signed-rank test for matched pairs. In comparison with their baseline values, the postoperative measures of both limb deformity and limb shortening improved significantly in both analyzed groups (limb deformity: Ilizarov method *Z* = 6.96, *p* < 0.001, *r* = 0.57; classic method *Z* = 2.90, *p* < 0.001, *r* = 0.28) (limb shortening: Ilizarov method *Z* = 6.42, *p* < 0.001, *r* = 0.52; classic method *Z* = 2.81, *p* = 0.005, *r* = 0.28).

The effect size in the Ilizarov group was larger both in terms of reducing both limb deformity and shortening (it is worth noting, however, that the Ilizarov treatment was used in patients with higher baseline values of both these parameters).

The ASAMI bone scores achieved in the experimental group were excellent in 67 cases, good in 7 cases, and poor in 1 case. The ASAMI functional scores were excellent in 48 cases, good in 26 cases, and poor in 1 case.

## Discussion

Despite the fact that tibial fracture healing disturbances are common in clinical practice, their treatment poses a significant challenge [7–9]. Most pseudarthroses are a result of inadequate bone-fragment stabilization and inadequate perfusion, which may later lead to infection and loss of bone tissue. The relevant literature contains reports on a number of surgical techniques (including plastic and reconstructive surgery techniques) that can be classified as limb-sparing procedures. These include extensive excision of nonviable soft tissues and sequestra, as well as the use of autologous bone grafts and free tissue flaps [3, 5, 12]. Moreover, advances in the development of orthopedic implants have helped achieve adequate bone-fragment stabilization, thus reducing the risk of blood vessel damage.

Pseudarthrosis of the tibia can be stabilized with the use of external fixators, bone plates, or intramedullary nails [2, 17–20]. There are no large population studies evaluating treatment outcomes in aseptic pseudarthrosis of the tibia and comparing the Ilizarov method with internal osteosynthesis. Apart from bone-fragment stabilization, the Ilizarov method provides bone-fragment compression or distraction, limb distraction, and bone realignment (in the case of concomitant shortening and deformity).

Binod et al. presented the results of pseudarthrosis treatment via a modified Judet approach in a group of 35 patients. Bone union was achieved in 100% of patients after a mean of 8.24 months [2]. Megas et al. reported the results of 50 patients with nonunion of the tibia being treated with intramedullary nails placed with the use of drilling. Bone union was achieved in 100% of cases; the mean treatment duration was 6 months [17]. Tsang analyzed the effectiveness of exchange nailing in patients with pseudarthrosis of the tibia and achieved union in 69% of cases. The median time to union was 8.7 months [19]. Elster achieved bone union in 138 out of 172 patients (80.2%) exposed to extracorporeal shock wave therapy (ESWT) over a mean period of 4 months [21]. Harshwal described the results of pseudarthrosis treatment with a mono-lateral external fixator. Bone union was achieved in 91.9% of cases after a mean period of 5 months [20]. Garnavos reviewed the literature on the techniques promoting bone healing in the case of tibial fractures, without the need to remove the inserted intramedullary nails. This review presented the effectiveness of various nonsurgical and surgical techniques [22]. One of such techniques is the Ilizarov method, which is a method used worldwide, particularly in the case of nonunion with an accompanying infection or extensive loss of bone tissue [14, 23–27].

The examples presented above show that there is no single ideal treatment method for pseudarthrosis of the tibia. Treatment success depends largely on identifying the factors responsible for nonunion and selecting the treatment method appropriate for the specific pathological mechanism.

In the group treated with the Ilizarov method, 100% of patients achieved bone union. The median time to union was 203 days, with the median of 192 days for patients with hypertrophic pseudarthrosis and 301 days for patients with atrophic pseudarthrosis. In the control group, bone union was achieved in 51% of patients; the Ilizarov group and the control group differed in terms of the median time to union (203 days and 271 days, respectively); however, this difference reached only borderline significance (*p* < 0.091). In the experimental group, there were 67 excellent, 7 good, and 1 poor ASAMI bone scores; and 48 excellent, 26 good, and 1 poor ASAMI functional scores. This is another piece of evidence supporting a high effectiveness of the Ilizarov method in treating bone nonunion. This effectiveness is likely due to low invasiveness of the surgical technique; stable, multi-planar structure of the fixator; and the fact that it allows early patient mobilization with full weight-bearing, which accelerates bone remodeling.

**Fig. 9:**
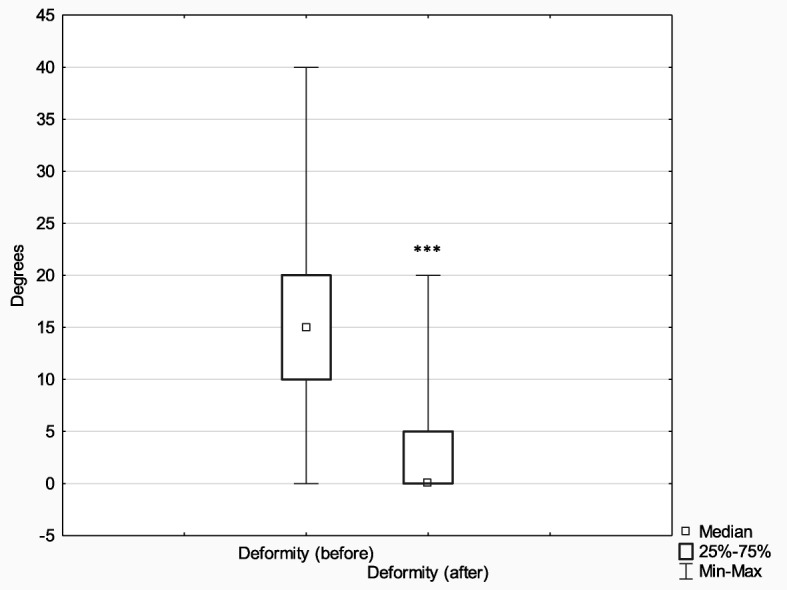
Median extent of leg deformity before and after treatment with the Ilizarov method; ***p ≤ 0.001

**Fig. 10:**
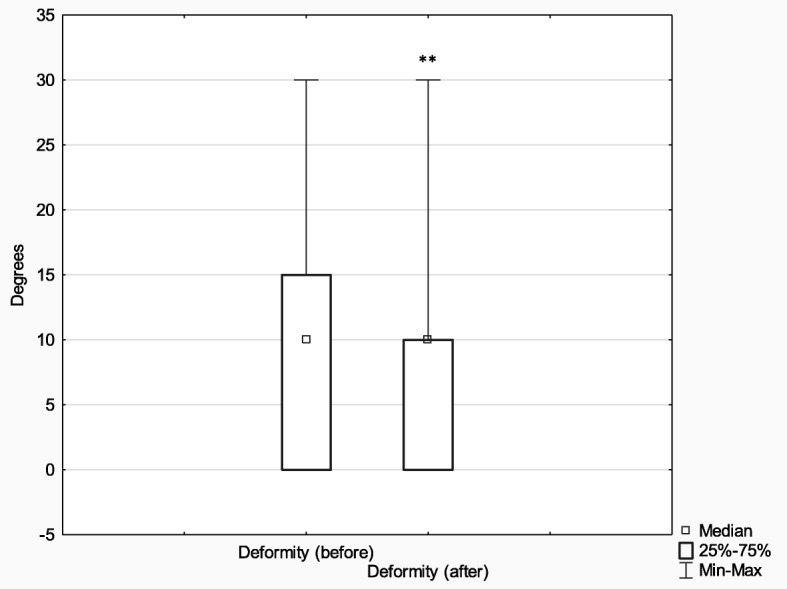
Median leg deformity before and after treatment with the classic method; **p ≤ 0.01

**Fig. 11:**
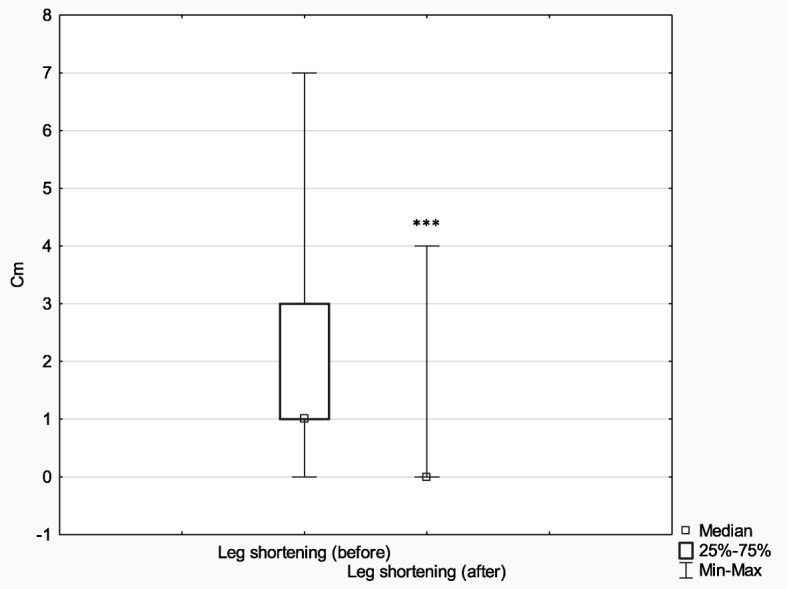
Median leg shortening before and after treatment with the Ilizarov method; ***p ≤ 0.001

**Fig. 12:**
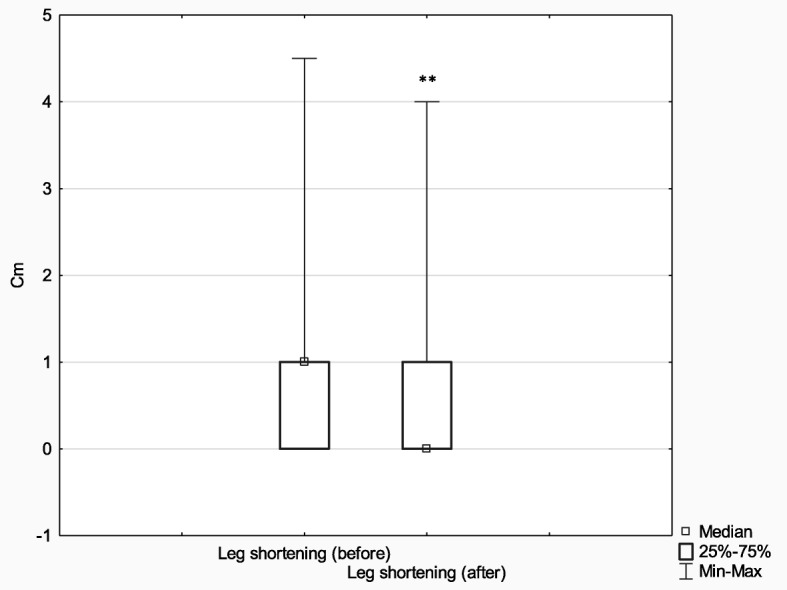
Median leg shortening before and after treatment with the classic method; **p ≤ 0.01

The initial extent of leg deformity and shortening was corrected in both groups; however, the effect size in patients treated with the Ilizarov method was larger. It should be noted that the Ilizarov group patients had greater initial leg deformity and shortening than control patients. More detailed results are presented in Figs. 9, 10, 11 and 12.

We observed no significant difference in terms of time to union between the group of patients with at least one risk factor for disturbance in fracture healing and the group with no risk factors. The following risk factors were considered: diabetes mellitus, corticosteroid therapy, smoking, alcohol dependence, and advanced lower-extremity vascular disease. The lack of significant difference suggests that the Ilizarov method should be recommended particularly in patients at risk of disturbance in fracture healing.

The most common complication observed in our study population during treatment with an Ilizarov fixator was Kirschner wire pin tract infection. Such infections typically respond well to topical antiseptics and oral antibiotic therapy in an outpatient setting. Deep infections involving soft tissues and bone require hospitalization, surgical debridement, and Kirschner wire replacement, which significantly lengthen the healing process (median, 189.0 days vs. 248.5 days). Sometimes, peri-implant infections lead to poor treatment outcomes [28–31], hence, the immense importance of a close cooperation between the patient and the treating team, regular follow-up visits, and adherence to doctor’s recommendations. The Ilizarov method is not recommended in persons who are obese, mentally ill, or addicted to psychoactive substances.

In summary, our study demonstrated a high effectiveness of the Ilizarov method in the treatment of aseptic posttraumatic pseudarthroses of the tibia. The Ilizarov method seems to be worth considering in all cases where either the patient or the nature of injury is associated with additional risk factors and whenever there is a need for leg deformity correction or leg elongation. The success of treatment depends on thorough preoperative planning, postoperative rehabilitation, and a close cooperation between the patient and the attending physician.

## Conclusions

1. The Ilizarov method is characterized by high effectiveness in the treatment of disturbances in tibial fracture healing. This method yields good treatment outcomes even in patients with risk factors for impaired fracture healing.
2. The time to union in pseudarthrosis of the tibia treated via the Ilizarov method is comparable with that achieved with intramedullary nailing.
3. The Ilizarov method offers a greater extent of correction in posttraumatic deformities and helps better correct posttraumatic limb shortening in comparison to the results achieved with internal osteosynthesis methods.
4. ASAMI functional scores are consistent with radiographic evidence (ASAMI bone scores).

## Data Availability

Not applicable

## Abbreviations

ASAMI: Association for the Study and Application of Methods of Ilizarov
LCP: Locking compression plate
ESWT: Extracorporeal shock wave therapy
